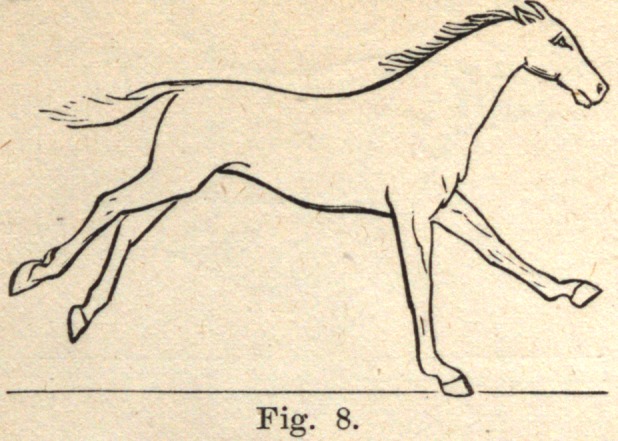# The Horse in Motion, as Shown by Instantaneous Photography

**Published:** 1882-04

**Authors:** 


### REVIEWS.

The Horse in Motion, as Shown by Instantaneous Photography, with a Study on Animal Mechanics, Founded on Anatomy and the Revelations of the Camera, in which is Demonstrated the Theory of Quadrupedal Locomotion. By J. D. B. Stillman. Executed and published under the auspices of Leland Stanford. Boston: James R. Osgood & Co.

Messrs. Osgood & Co., of Boston, have just published a handsome and interesting quarto volume, entitled "The Horse in Motion," prepared at an outlay of over $50,000 by Governor Stanford, and years of scientific experiments and investigation by Mr. Muybridge at the Palo Alto stud farm of the Governor. It contains a series of photographs of the horse while in motion, taken by means of twenty-four cameras, placed in line one foot apart, capable of producing an impression by the exposure of one five thousandth part of a second, so great was the sensitiveness of each camera. By means of these the position of each part of the horse's stride while running was obtained with scientific exactness, and the results of these experiments prove that the hitherto prevailing notions regarding the modes of motion of the horse were all wrong. The theory generally accepted was that the horse when running lifted his fore feet, and with an impulse from his haunches plunged into the air, alighting on his fore legs. The photographs show that this is entirely different, the hind feet, which are the chief instruments of propulsion, being the first to leave the ground, one fore foot being used as a sort of lever to lift the whole body, and then employed as a leaping pole to propel the body forward. We give sketches of the cuts that show the movement of a running horse:

Fig. 1.

**Fig. 1** gives the position of the animal in readiness to start.

Fig. 2.

Fig. 2 represents the beginning of movement, with the hind legs up in the air, the left fore foot upon the ground, nearly under the centre of gravity, while the right fore foot is bent upward.

Fig. 3.

Fig 3 shows the body carried forward, so that it can no longer be supported by the forelegs.

Fig. 4.

Fig. 4 shows all the feet off the ground, and the horse completely in the air. There is now an opportunity, if the horse wishes, to change his feet in the gallop.

Fig. 5.

The first check to the descent of the centre of gravity is given by one of the hind legs, as shown in Fig. 5.

Fig. 6.

Fig. 6 represents both hind legs upon the ground, the body moving forward and requiring more advanced support.

Fig. 7.

Fig. 7. The right hind leg has given its final propulsive impulse, the left hind leg has passed the perpendicular, and is no longer in a position to give much aid as a supporter to the centre of gravity; but the right forefoot has reached the ground and takes its position as a supporter of the weight of the body, dividing the burden with the left hind leg still upon the ground.

Fig. 8.

Fig. 8. The right fore leg is now taking the entire weight of the body; the left hind foot is clear of the ground, the muscles of the near hind leg are exerted, and from them the body receives a new impetus, and is carried forward, upheld by the off fore leg.

The scientific world is deeply indebted to Governor Stanford for this useful work, in the preparation of which no expense has been spared, and veterinarians especially will read with interest and profit Dr. Stillman's clear and succinct explanation of the muscles of the horse.

## Figures and Tables

**Fig. 1. f1:**
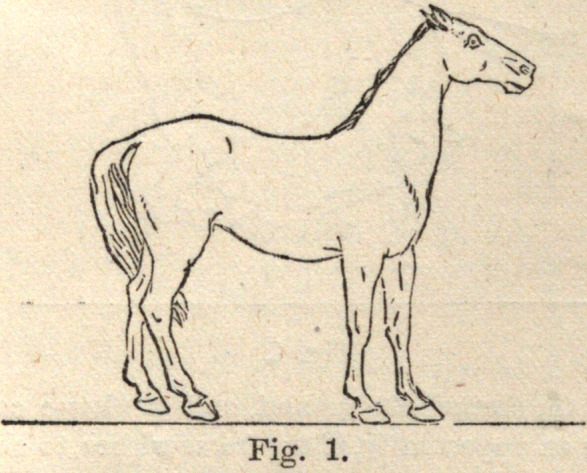


**Fig. 2. f2:**
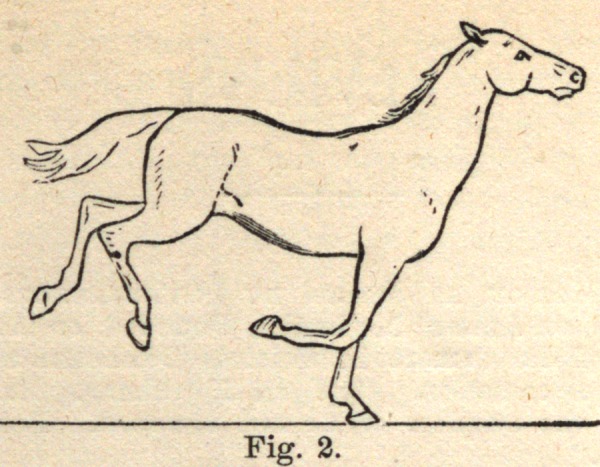


**Fig. 3. f3:**
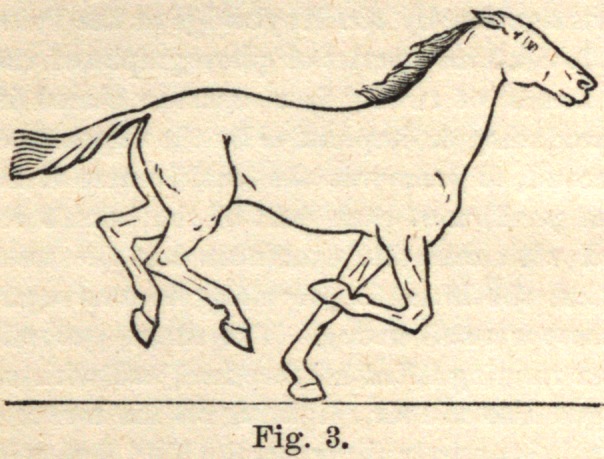


**Fig. 4. f4:**
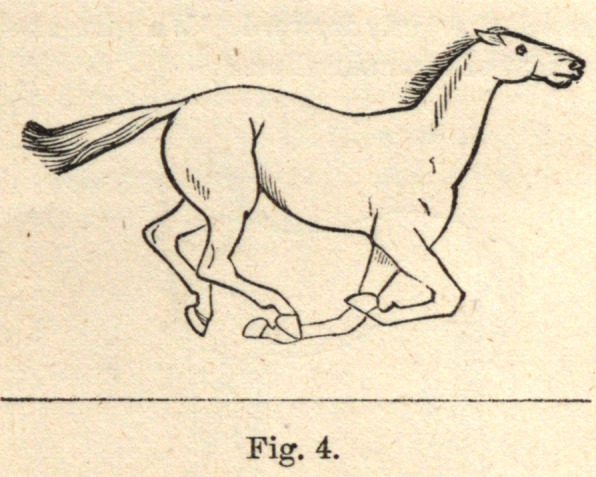


**Fig. 5. f5:**
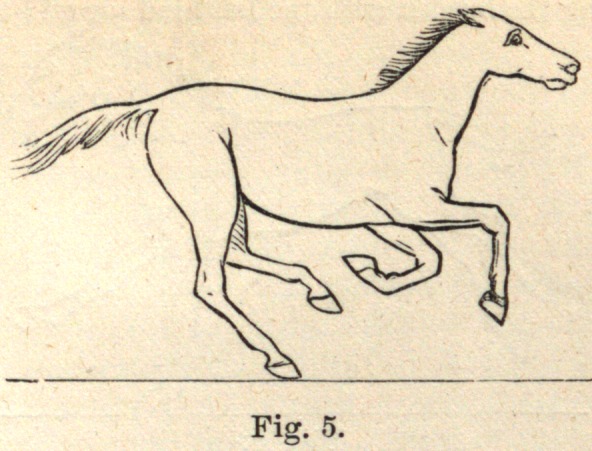


**Fig. 6. f6:**
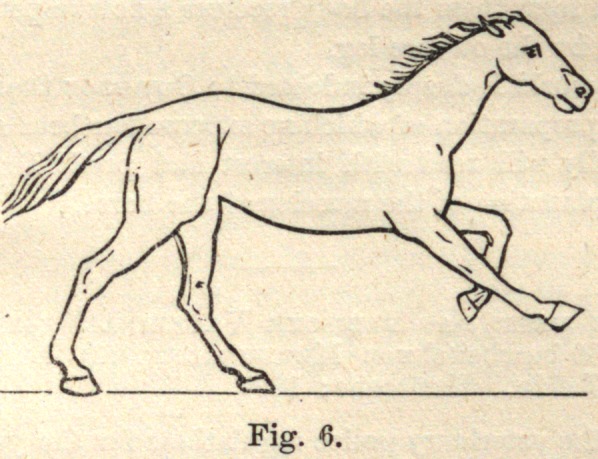


**Fig. 7. f7:**
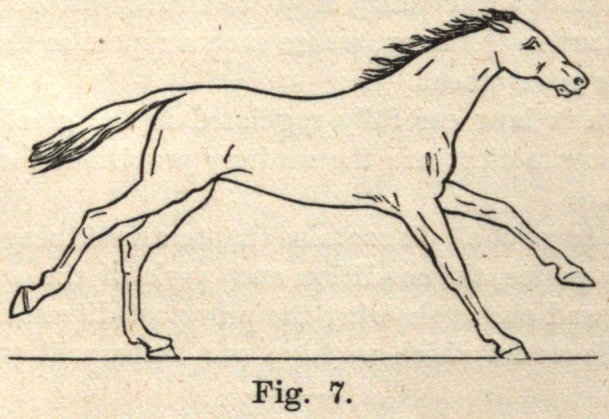


**Fig. 8. f8:**